# Netrin-1 functions as a suppressor of bone morphogenetic protein (BMP) signaling

**DOI:** 10.1038/s41598-021-87949-7

**Published:** 2021-04-21

**Authors:** Ahmad Abdullah, Carl Herdenberg, Håkan Hedman

**Affiliations:** 1 Department of Radiation Sciences, Oncology, Umeå University, 90187 Umeå, Sweden; 2 Oncology Research Laboratory, NUS M31, 90185 Umeå, Sweden

**Keywords:** Transforming growth factor beta, Kinases, Oncogene proteins, Cancer, Cell death, Cell signalling, Mechanisms of disease, Bone development, Embryogenesis, Neurogenesis, Pattern formation, Stem cells

## Abstract

Netrin-1 is a secreted protein that is well known for its involvement in axonal guidance during embryonic development and as an enhancer of cancer cell metastasis. Despite extensive efforts, the molecular mechanisms behind many of the physiological functions of netrin-1 have remained elusive. Here, we show that netrin-1 functions as a suppressor of bone morphogenetic protein (BMP) signaling in various cellular systems, including a mutually inhibitory interaction with the BMP-promoting function of leucine-rich repeats and immunoglobulin-like domains (LRIG) proteins. The BMP inhibitory function of netrin-1 in mouse embryonic fibroblasts was dependent on the netrin receptor neogenin, with the expression level regulated by both netrin-1 and LRIG proteins. Our results reveal a previously unrecognized function of netrin-1 that may help to explain several of the developmental, physiological, and cancer-promoting functions of netrins at the signal transduction level.

## Introduction

Netrin-1 is a secreted protein that is encoded by the *NTN1* gene in humans and is expressed by many tissues and cell types [www.proteinatlas.org]. In mammals, the netrin protein family comprises five expressed members: netrin-1, netrin-3, netrin-4, netrin-G1, and netrin-G2^1^. Netrins play important roles in axon guidance via interactions with their receptors, which belong to the Unc-40 family, including deleted in colorectal carcinoma (DCC) and neogenin, and Unc-5 family, including UNC5A, UNC5B, UNC5C, and UNC5D^1–3^. Recently, Down syndrome cell adhesion molecule (DSCAM) and melanoma cell adhesion molecule (MCAM, also known as CD146 or MUC18) were also found to function as high-affinity netrin-1 receptors^3,4^; however, the biological significance of these interactions remains controversial. Recently, it has become clear that netrins play important roles not only in axon guidance but also in many other developmental and physiological processes.

During inner ear development in mice, *Ntn1* reciprocally opposes both *leucine-rich repeats and immunoglobulin-like domains 3* (*Lrig3*)^5^ and *Bmp2*^6^. Netrin-1 also seems to play a role in bone remodeling, where it inhibits osteoblast differentiation^7^ and supports osteoclast differentiation^8^. Netrin-1 may also play a role in metabolic homeostasis and disease, as suggested by recent findings showing that the serum levels of netrin-1 are elevated in patients with impaired fasting glucose or type 2 diabetes^9,10^, that the expression of genes related to the netrin-1 signaling pathway is increased in blood from subjects with fatty liver^11^ and that at the cellular level, netrin-1 promotes the proliferation of adipose-derived stem cells^12^. Netrin-1 is often highly expressed in cancer tissues (see, for example, the Human Protein Atlas [www.proteinatlas.org]) and has been reported to promote tumorigenesis in a variety of systems^13^. For example, forced netrin-1 overexpression in the mouse intestine results in hyperplasia and adenoma formation^14^, autocrine netrin-1 expression is implicated in the progression of experimental inflammatory bowel disease to colorectal cancer^15^, autocrine netrin-1 expression promotes experimental breast cancer metastasis^16^, and in glioma, netrin-1 promotes cancer cell invasion and stemness^17^. Netrin-1 has also been proposed to enhance angiogenesis^18^. Recently, colon and lung cancer-associated fibroblasts have been shown to produce netrin-1, which enhances the tumorigenicity of cocultured lung cancer and colorectal cancer cells^19^. In some contexts, netrin-1 protects cells against apoptosis, and it has been suggested that the netrin-1 receptors DCC, neogenin, UNC5A, UNC5B, UNC5C, and UNC5D function as ‘dependence receptors’, i.e., receptors that initiate apoptotic signaling in the absence of ligands^20,21^. Intriguingly, the molecular mechanisms behind many of the developmental, physiological, and tumor-promoting functions of netrin-1 remain poorly characterized.

Given the many prominent developmental and physiological functions of netrin-1, it is important to elucidate the molecular mechanisms by which these functions are executed. Here, we show that netrin-1 negatively regulates bone morphogenetic protein (BMP) signaling in different cellular contexts. This discovery might help to explain many previous observations regarding the physiological and pathological functions attributed to this pleiotropic protein.

## Materials and methods

### Reagents and antibodies

Human recombinant BMP4 (#120-05), BMP6 (#120-06), BMP9 (#120-07), and mouse noggin (#250-38) were purchased from PeproTech Nordic (Stockholm, Sweden). Human recombinant netrin-1 (#6419-N1) was obtained from Bio-Techne Ltd. (Abingdon, UK). The antibodies used in this study are listed in Table S1.

### Cell lines and cell culture

Mouse embryonic fibroblast (MEF) lines established in our laboratory according to the 3T3 protocol^22^ were cultured in Dulbecco’s modified Eagle’s medium (DMEM) (Sigma-Aldrich Sweden AB, Stockholm, Sweden) supplemented with 10% fetal bovine serum (FBS) (Fisher Scientific GTF AB, Gothenburg, Sweden), MEM-nonessential amino acids (Fisher Scientific GTF AB), 50 μM 2-mercaptoethanol (Sigma-Aldrich Sweden AB), and 50 μg/ml gentamicin (Invitrogen, Fisher Scientific GTF AB). The human ovarian cancer cell lines Ovcar-3 and Ovsaho (cell number: JCRB1046) were obtained from American Type Culture Collection (ATCC) (Manassas, VA, USA) and Japanese Collection of Research Bioresources (Osaka, Japan), respectively, and they were maintained in RPMI 1640 (Sigma-Aldrich Sweden AB) containing 20% FBS with 10 μg/ml bovine insulin (Sigma-Aldrich Sweden AB, cat. #I0516) and 10% FBS, respectively. Human acute lymphoblastic leukemia cell lines 697 and RCH-ACV were obtained from the Leibniz Institute DSMZ-German collection of microorganisms and cell cultures (Braunschweig, Germany) and maintained in RPMI 1640 supplemented with 10% FBS, 22 mg/ml sodium pyruvate (Sigma-Aldrich Sweden AB, #S8636), and 50 μg/ml gentamicin. Human glioblastoma cell lines TB101 and TB107 were previously established in our laboratory^23,24^ and maintained in F12 medium (Invitrogen, Fisher Scientific GTF AB, #11320) supplemented with penicillin–streptomycin (Invitrogen, Fisher Scientific GTF AB, #15140, 1:100), 20 ng/ml EGF (R&D Systems Europe Ltd., Abingdon, UK, #236-EG), 20 ng/ml bFGF (#233-FB, R&D Systems Europe Ltd.), B27 (Invitrogen, Fisher Scientific GTF AB, #17504-044), and N2 (Invitrogen, Fisher Scientific GTF AB, #17502-048) and cultured in laminin-coated (Sigma-Aldrich Sweden AB, #11243217001) flasks for maintenance and in laminin-coated 12-well plates for experiments. The ATDC5 cell line was obtained from Sigma-Aldrich Sweden AB (#99072806-1VL) and maintained in DMEM:Ham′s F12 (1:1) containing 2 mM glutamine and supplemented with 5% FBS and 50 μg/ml gentamicin. The HEK293, A375, HCT116, LoVo, MDA-MB-451, and T-47D cell lines were obtained from colleges and authenticated through short tandem repeat profiling via Eurofins Genomics Germany GmbH (Ebersberg, Germany) or ATCC. HEK293, A375, HCT116, and LoVo cells were cultured in DMEM supplemented with 10% FBS and 50 μg/ml gentamicin. MDA-MB-451 and T-47D cells were cultured in RPMI 1640 with 10% FBS and 50 μg/ml gentamicin. All the cell lines were maintained at 37 °C in a humidified atmosphere containing 5% CO_2_.

### Phospho Smad1/5 immunofluorescence assay

Cells were seeded at a density of 3,000 cells per well in a 96-well microtiter plate (#655090, Greiner Bio-One International GmbH, Monroe, NC, USA) coated with 0.1% bovine gelatin. After an overnight incubation, the cells were starved in serum-free medium for 1 h followed by the induction of BMP signaling using different concentrations of BMP4, BMP6, or BMP9. After an hour of BMP treatment, the cells were washed with phosphate-buffered saline (PBS) and fixed with 4% formaldehyde. For the cross-titration studies, *Ntn1*^−/−^, *Lrig*-null MEFs with Tet-On inducible *LRIG1-FLAG or LRIG3-FLAG* alleles were used. The cells were treated with different concentrations of doxycycline overnight. The next day, the cells were serum-starved for an hour followed by the addition of 5 ng/ml BMP4 with different amounts of netrin-1. After 1 h of incubation, the cells were washed with PBS and fixed with 4% formaldehyde. To investigate the kinetics of netrin-1 inhibition, 0.25 μg/ml netrin-1 was added to wild-type MEFs at different timepoints before, with, or after the addition of BMP4. Sixty minutes after the addition of BMP4, the cells were washed with PBS and fixed with 4% formaldehyde. Then, the pSmad assay was conducted as described previously^25^.

### RNA extraction and quantitative RT-PCR

RNA extraction was carried out using a PureLink RNA Mini Kit (Invitrogen, Fisher Scientific GTF AB) with PureLink DNase (Invitrogen, Fisher Scientific GTF AB) treatment. A TaqMan assay was used to quantify the gene expression of *Id1* (Mm00775963_g1, Fisher Scientific GTF AB, #4331182), and normalization was performed using the reference gene *RN18S* as an internal control as described previously^26^. Data acquisition was performed using a CFX96 system C1000 thermal cycler (Bio-Rad Laboratories AB).

### *Ntn1* and *Neo1* gene ablations

*Lrig*-triple FLOXed MEFs (*Lrig1*^*fl/fl*^*;Lrig2*^*fl/fl*^*;Lrig3*^*fl/fl*^) or *Lrig*-null MEFs (*Lrig1*^−/−^*;Lrig2*^−/−^*;Lrig3*^−/−^) with inducible *LRIG1* or *LRIG3* alleles^26^ were transfected using Fugene6 transfection reagent (Promega Biotech AB, Nacka, Sweden), as previously described^26^, using the CRISPR/Cas9 HDR-mediated knockout kit KN311288RB (*Ntn1*) or KN310902RB (*Neo1*) from OriGene Technologies (BioNordika Sweden AB, Solna, Sweden) and a donor plasmid with a Blasticidin S resistance gene together with either a CRISPR/Cas9 plasmid guide-RNA for the *Ntn1* or *Neo1* gene or a scramble control plasmid. Forty-eight hours after transfection, 10 μg/ml blasticidin S (Gibco, Fisher Scientific GTF AB) was added to the cells. After two weeks of selection with blasticidin S, the resistant MEFs were cloned, yielding three *Ntn1* knockout MEF clones together with four scramble wild-type control MEF clones and four *Neo1* knockout MEF clones together with four scramble wild-type control MEF clones. The *Ntn1* knockout MEF clones were then transduced with adenovirus carrying either Cre recombinase or control to create *Lrig*-null MEFs as previously described^26^.

### Western blot analysis

Cells were seeded in 6- or 12-well plates the day before stimulation with netrin-1 and BMP4. On the day of stimulation, the cells were starved in FBS-free media for an hour prior to stimulation with 10 ng/ml BMP4 and 0.25 μg/ml netrin-1. After one hour of stimulation, the cells were washed with PBS and lysed with lysis buffer (Invitrogen, Fisher Scientific GTF AB) containing a protease inhibitor cocktail (Roche Diagnostics Scandinavia AB, Bromma, Sweden) and the phosphatase inhibitor PhosSTOP (Roche Diagnostics Scandinavia AB). The cell lysates were kept on ice for 30 min and centrifuged at 20,800×g for 10 min at 4 °C. The clear supernatant was subjected to SDS-PAGE using 3–8% Tris–acetate gels (#EA03752, Invitrogen, Fisher Scientific GTF AB) followed by transfer onto nitrocellulose or PVDF membranes (Bio-Rad Laboratories AB, Solna, Sweden). The membranes were blocked using Odyssey blocking buffer (LI-COR Biosciences GmbH, Bad Homburg, Germany). The blocked membranes were then incubated with the primary antibodies at room temperature for an hour. Thereafter, the membranes were washed three times with TBST (20 mM Tris, 150 mM NaCl, 0.1% Tween 20), incubated with the fluorescent secondary antibodies for an hour at room temperature and then washed again three times with TBST. The fluorescent bands were analyzed using the Odyssey CLx imaging system (LI-COR Biosciences GmbH).

### Adipogenesis assay

The adipogenesis assay has been described elsewhere^26^. Briefly, the MEFs were treated with an adipogenic cocktail with or without 0.3 μg/ml netrin-1 for 9 days. Then, the cells were fixed, and the adipocytes were stained using Oil Red O (Sigma-Aldrich Sweden AB). The Oil Red O-stained cells were quantified in a Spectramax i3x plate reader (Molecular Devices, San Jose, CA, USA) using Softmax Pro 7 software (Molecular Devices) as described elsewhere^26^.

### Alkaline phosphatase activity assay

For the alkaline phosphatase (ALP) activity assay, 3,000 ATDC5 cells were seeded per well in a 96-well microtiter plate and cultured overnight. The next day, the culture medium was replaced with DMEM:Ham′s F12 (1:1) containing 2 mM glutamine and supplemented with 2% FBS, 2 µg/ml heparin (Sigma Aldrich Sweden AB, #H3149), and 50 μg/ml gentamicin. BMP4, netrin-1, or noggin was added at different concentrations as indicated in the Results section. After 72 h of incubation, the cells were washed with PBS, and the ALP activity assay was carried out using a colorimetric kit (Abcam, Sweden, #ab83369) according to the manufacturer’s guidelines.

### Statistical analyses

All the data are presented as the averages of three or more independent experiments with standard deviations. Statistical comparisons were performed using a two-sided Student’s *t*-test or a one-way analysis of variance (ANOVA) with multiple comparisons using Dunnett’s test (GraphPad Prism, version 5.0), and a p-value of less than 0.05 was considered statistically significant.

## Results

### Exogenously added netrin-1 inhibits BMP signaling in wild-type MEFs

**Figure 1:**
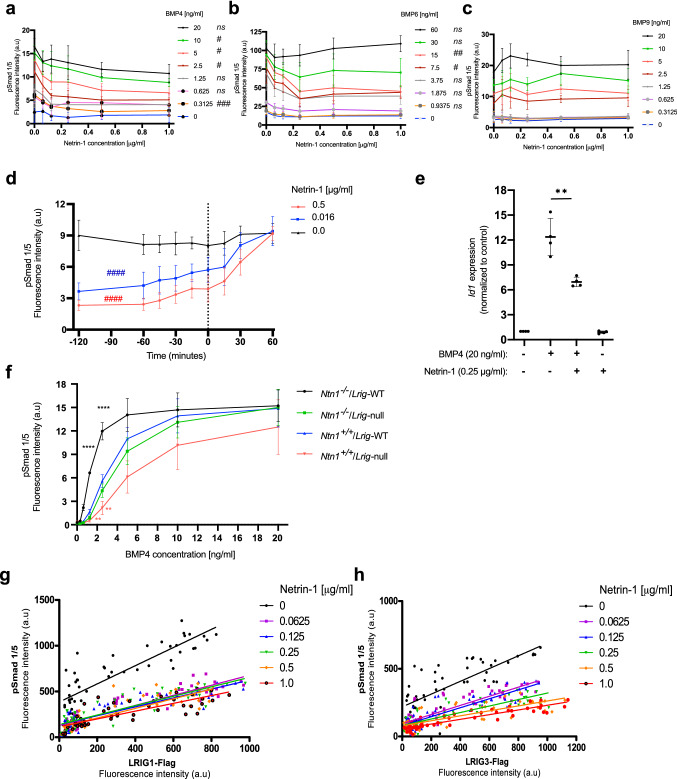
Netrin-1 inhibits BMP signaling in MEFs. (**a–c**) Wild-type MEFs were starved in serum-free medium and then treated with various concentrations of BMP4, BMP6, or BMP9 together with various concentrations of netrin-1 for an hour. Then, BMP signaling was assessed via the analysis of nuclear pSmad1/5 levels. The graphs show the average means with error bars showing the standard deviations of three independent experiments performed in duplicate (one-way ANOVA: ^ns^p ≥ 0.05; ^#^p < 0.05; ^##^p < 0.01; ^###^p < 0.001). (**d**) Wild-type MEFs were treated with various concentrations of netrin-1 at different times before, with, or after the addition of BMP4 [5 ng/ml] to the wells. One hour after the addition of BMP4, BMP signaling was assessed via the analysis of nuclear pSmad1/5 levels. The x-axis shows the time at which netrin-1 was added relative to the addition of BMP4: negative values indicate the addition of netrin-1 before the addition of BMP4, ‘0′ indicates the simultaneous addition of netrin-1 and BMP4, and positive values indicate the addition of netrin-1 after the addition of BMP4. The graph shows the average means with the standard deviations of four independent experiments performed in duplicate (one-way ANOVA: ^####^p < 0.0001). (**e**) *Id1* mRNA expression levels in response to BMP4 and netrin-1. MEFs were treated, or not, with 20 ng/ml BMP4 and/or 0.25 μg/ml netrin-1 for x hours followed by analysis of RNA expression levels through real-time qRT-PCR. Shown are the *Id1* levels normalized to the *Rn18s* levels. The graph represents the average means with standard deviations of four independent experiments performed in duplicate (Student’s *t*-test: **p < 0.01; ***p < 0.001). (**f**) MEFs of different *Ntn1* and *Lrig* genotypes (*Ntn1*^+*/*+^ or *Ntn1*^−/−^ and *Lrig*-wild-type or *Lrig*-null [*Lrig1*^−/−^*;Lrig2*^−/−^*;Lrig3*^−/−^], respectively) were treated with various concentrations of BMP4, and BMP signaling was assessed via analysis of the nuclear pSmad1/5 levels. The graph shows the means with the standard deviations of four *Ntn1*^+*/*+^*;Lrig*-wild-type or *Ntn1*^+*/*+^*;Lrig*-null biological replicates and three *Ntn1*^−/−^*;Lrig*-wild-type or *Ntn1*^−/−^*;Lrig*-null biological replicates determined through three independent experimental repeats. The error bars represent the standard deviations (Student’s *t*-test: **p < 0.01; ****p < 0.0001. Colors of asterisks represent the respective genotype, according to the graph symbols, compared to *Ntn1*^+*/*+^*;Lrig-WT*). (**g, h**) Cross-titration of netrin-1 and LRIG1 (**g**) or LRIG3 (**h**). LRIG1- or LRIG3-inducible *Lrig*-null and *Ntn1*^−/−^ MEFs were treated with different concentrations of doxycycline (between 0 and 1 μg/ml) to induce various levels of LRIG protein expression. BMP signaling was induced using 5 ng/ml BMP4 together with different amounts of netrin-1 (between 0 and 1 μg/ml) for an hour. The fluorescence response for the nuclear pSmad1/5 levels induced by BMP4 is plotted on the y-axis. The doxycycline-induced expression levels of FLAG-tagged LRIG1 or LRIG3 were measured and plotted on the x-axis. Each dot represents the quantification of pSmad1/5 and LRIG-FLAG from each measurement. The graphs show data obtained from three independent experiments performed in duplicate for each concentration of netrin-1 and doxycycline. The R^2^ values for the scattered plots are tabulated in Tables 1 and 2.

To investigate whether netrin-1 regulates BMP signaling in MEFs, netrin-1 was cross-titrated against BMP4, BMP6, or BMP9, and the BMP response was analyzed via a cellular phospho-Smad1/5 assay (Fig. 1a–c). Clearly, exogenously added netrin-1 inhibited BMP4- and BMP6-induced signaling in a dose-dependent manner but not BMP9-induced signaling. The IC_50_ for the inhibitory effect of netrin-1 at 2.5 ng/ml BMP4 was approximately 100 ng/ml (Fig. 1a). To investigate the kinetics of netrin inhibition, netrin-1 was added at different time points before, with, or after the initiation of BMP stimulation (Fig. 1d). There was a stronger inhibition the earlier netrin-1 was added to the cells; however, the addition of netrin-1 after the initiation of BMP4 stimulation also inhibited signaling. To investigate whether the observed inhibition of Smad1/5 phosphorylation translated into altered gene expression, BMP4-induced *Id1* expression was assessed through qRT-PCR (Fig. 1e). Obviously, the BMP4-induced expression of *Id1* was also inhibited by netrin-1.

**Table 1:** The coefficient of determination (r^2^) for the BMP4 response (pSmad1/5 level) against the LRIG1- FLAG expression levels at different netrin-1 concentrations as shown in Fig. 1g.

Netrin-1 [μg/ml]	0	0.0625	0.125	0.25	0.5	1.0
r^2^	0.7384	0.8220	0.7754	0.7473	0.7580	0.6596

**Table 2:** The coefficient of determination (r^2^) for the BMP4 response (pSmad1/5 level) against the LRIG3- FLAG expression levels at different netrin-1 concentrations as shown in Fig. 1h.

Netrin-1 [μg/ml]	0	0.0625	0.125	0.25	0.5	1.0
r^2^	0.5863	0.262	0.7716	0.7103	0.6208	0.8273

### Endogenously expressed netrin-1 functions as a relevant BMP signaling-modulator in MEFs and interacts with LRIG proteins in a mutually inhibitory manner

To investigate whether endogenously expressed netrin-1 affects BMP signaling in MEFs, we generated *Ntn1*-null (*Ntn1*^−/−^) MEFs (Fig. S1). Because *Lrig3* has been shown to interact genetically with *Ntn1*^5^, we generated *Ntn1*-null MEFs both on an *Lrig* wild-type background and on an *Lrig*-null (*Lrig1*^−/−^*;Lrig2*^−/−^*;Lrig3*^−/−^) background. Clearly, the *Ntn1*-null MEFs showed increased BMP4 sensitivity compared to the wild-type MEFs (Fig. 1f). The increased BMP sensitivity in *Ntn1*^−/−^ MEFs was not the result of increased Bmpr2, Acvr1, or Smad1 expression levels; rather, these signaling mediators showed insignificant trends toward lower expression levels (Fig. S2a–d). The Lrig1 and Lrig3 levels were not altered in *Ntn1*^−/−^ MEFs (Fig. S3a–d), nor were the netrin-1 levels altered in *Lrig*-null MEFs (Fig. S3e,f). *Lrig*-null MEFs displayed decreased BMP sensitivity compared to wild-type MEFs (Fig. 1f), as was recently shown elsewhere^26^. The MEFs with combined deficiency of *Ntn1* and *Lrig* (*Ntn1*^−/−^*;Lrig1*^−/−^*;Lrig2*^−/−^*;Lrig3*^−/−^) showed an intermediate BMP sensitivity that was comparable to the BMP sensitivity of the wild-type MEFs (Fig. 1f). This result showed that endogenously expressed netrin-1 is a relevant negative regulator of BMP signaling that balances the BMP-promoting activities of Lrig proteins in MEFs. To further investigate the mutually inhibitory interactions between netrin-1 and LRIG proteins, the pSmad1/5 response was analyzed in *LRIG1*- or *LRIG3*-inducible *Ntn1*^*-/*^;*Lrig1*^−/−^;*Lrig2*^−/−^;*Lrig3*^*-/–*^ MEFs (Fig. S1b,c) with various levels of LRIG protein induction that had been treated with BMP4 and different concentrations of netrin-1 (Fig. 1g,h). This experiment showed that netrin-1 was able to suppress BMP4 signaling in a dose-dependent manner, both in the absence and in the presence of the previously shown BMP4-promoting activities of LRIG1 and LRIG3, further documenting the mutually inhibitory interaction between netrin-1 and LRIG proteins on BMP4 signaling strength.

### Netrin-1 suppresses BMP signaling in various cell types

**Figure 2:**
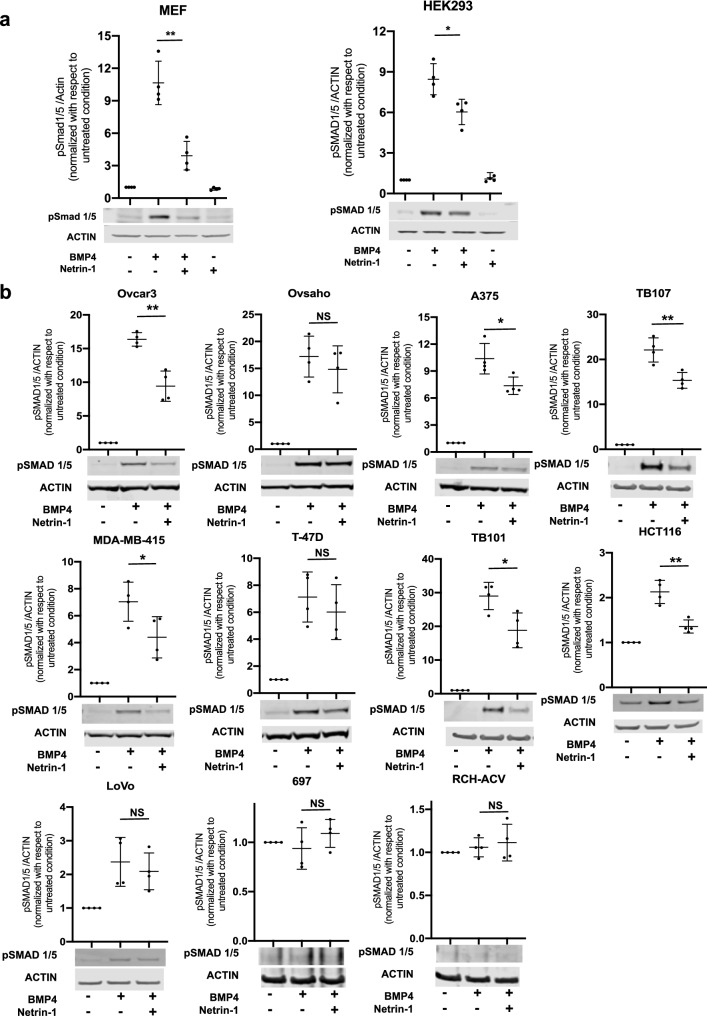
Netrin-1 inhibits BMP signaling in various mammalian cell lines. Western blot analysis showing the inhibitory effect of netrin-1 on the BMP4-induced pSmad1/5 levels in cell lines of different origins. (**a**) MEFs and human embryonic kidney (HEK293) cells. (**b**) Human ovarian cancer (Ovcar3 and Ovsaho), malignant melanoma (A375), breast cancer (MDA-MB-415 and T-47D), glioblastoma (TB101 and TB107), colorectal carcinoma (HCT116 and LoVo), and acute lymphoblastic pre-B cell leukemia (697 and RCH-ACV). Representative Western blots of pSMAD1/5 and actin after the cells were treated with BMP4 and netrin-1 are shown together with the quantifications of the pSMAD1/5/actin ratios. Uncropped blots are shown in Fig. S4. Shown are the means and individual values of four independent experiments (Student’s *t*-test: ^NS^p ≥ 0.05; *p < 0.05; **p < 0.01).

To investigate the generality of the observed netrin-1-induced suppression of BMP signaling, a panel of mammalian cell lines consisting of an MEF line together with twelve human cell lines was evaluated to determine their sensitivity to netrin-1-mediated inhibition. The human cell lines were chosen to represent different tissues of origin. The cells were treated with BMP4 in the presence or absence of netrin-1 for 60 min, and the pSMAD1/5 levels were analyzed by Western blot (Fig. 2). MEFs and HEK293 cells were also treated with netrin-1 alone (Fig. 2a); however, because treatment with netrin-1 alone had no effect on pSmad1/5 levels in MEFs or HEK293 cells, ‘netrin-1 only’ treatment was not assessed in the other cell lines (Fig. 2b). The pre-B acute lymphocytic leukemia cell lines 697 and RCH-ACV did not respond to BMP4 treatment with increased pSMAD1/5 levels and thus could not be evaluated for possible inhibition by netrin-1. Of the eleven cell lines that responded to BMP4, eight were significantly inhibited by netrin-1, including the MEF line, the ovarian cancer cell line Ovcar-3, the malignant melanoma cell line A375, the breast cancer cell line MDA-MB-415, the human embryonic kidney cell line HEK293, the glioblastoma cell lines TB101 and TB107, and the colorectal cancer cell line HCT116 (Fig. 2). Thus, netrin-1 inhibited BMP signaling in a general manner, affecting mouse and human cells of various tissues of origin.

### Netrin-1-induced inhibition of BMP signaling in MEFs is dependent on neogenin

**Figure 3:**
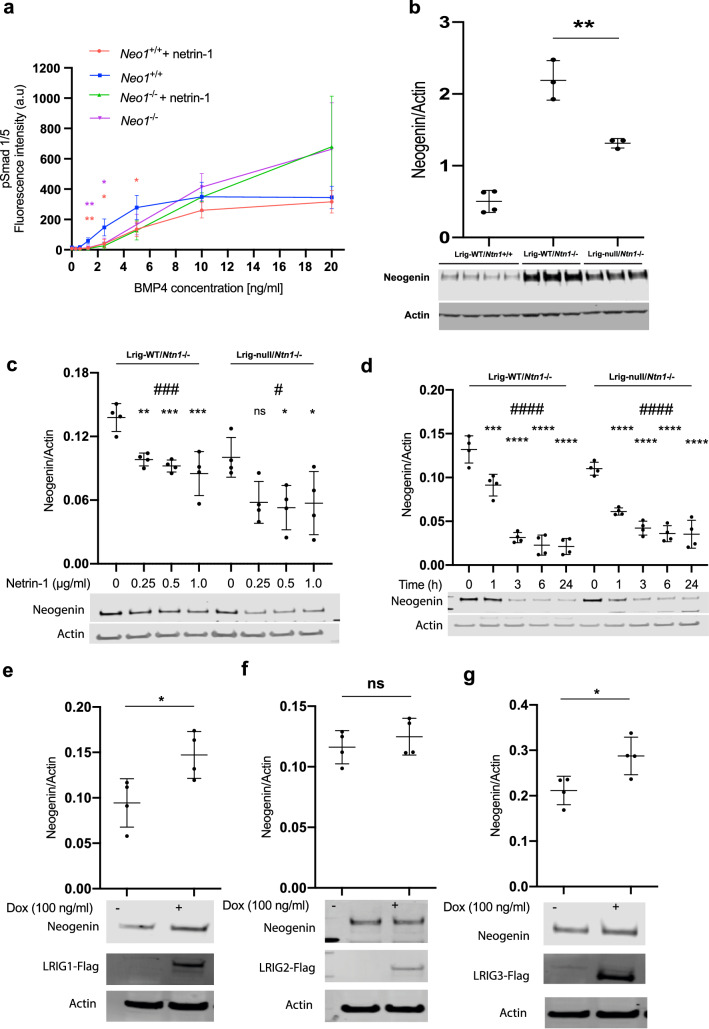
Neogenin is required for netrin-1-induced inhibition of BMP signaling, and its expression levels are regulated by netrin-1 and LRIG proteins. (**a**) Effects of netrin-1 on BMP signaling in *Neo1* wild-type (*Neo1*^+*/*+^) and *Neo1*-deficient (*Neo1*^−/−^) MEFs. The cells were treated with different concentrations of BMP4 in the presence or absence of 0.25 μg/ml netrin-1. Signaling was analyzed using a pSmad1/5 assay. The graph shows the average means with the standard deviations of four biological replicates. (**b**) Neogenin expression levels in MEFs of different genotypes (*Ntn1*^+*/*+^ or *Ntn1*^−/−^ and *Lrig*-wildtype or *Lrig*-null [*Lrig1*^−/−^*; Lrig2*^−/−^*; Lrig3*^−/−^]). Neogenin and actin levels were analyzed by Western blotting. The graph represents the average means with standard deviations of band intensity ratios (neogenin/actin) from four *Ntn1*^+*/*+^*;Lrig*-wild-type biological replicates and three *Ntn1*^−/−^*;Lrig*-wild-type or *Ntn1*^−/−^*;Lrig*-null biological replicates that were determined with three independent experiments. (**c, d**) Concentration-dependent (**c**) and time-dependent (**d**) effects of netrin-1 treatment on neogenin expression levels in *Ntn1*^−/−^;*Lrig*-wild-type and *Ntn1*^−/−^;*Lrig*-null MEFs. Representative blots and graphs showing the average means with standard deviations of four independent experiments are shown (one-way ANOVA: ^#^p < 0.05; ^###^p < 0.001; ^####^p < 0.0001. Post-hoc test with multiple comparisons [compared to netrin-1, 0 ng/ml] adjusted p-value: ^ns^p ≥ 0.05; *p < 0.05; **p < 0.01; ***p < 0.001; ****p < 0.0001). (**e–g**) Effects of induced expression of LRIG1 (**e**), LRIG2 (**f**), and LRIG3 (**g**) on neogenin expression levels. LRIG expression was induced through the treatment of LRIG-inducible MEFs with 100 ng/ml of doxycycline, overnight. Neogenin and actin levels were analyzed using Western blotting. Uncropped blots are shown in Fig. S7. Representative blots and graphs representing average means with standard deviations of four independent experiments are shown. (Student’s *t*-test; *, p < 0.05; **, p < 0.01; ***, p < 0.001. Colors of asterisks in (**a**) represent the respective genotype, according to the graph symbols, compared to *Neo1*^+*/*+^).

To address the role of neogenin in netrin-1-induced inhibition of BMP signaling in MEFs, *Neo1* was inactivated through CRISPR/Cas-9 (Fig. S5). The resulting *Neo1*^−/−^ MEFs showed reduced sensitivity to low concentrations of BMP4 (Fig. 3a). The Lrig1, Lrig3, and netrin-1 levels were unaltered in the *Neo1*-deficient MEFs (Fig. S5a–d). Intriguingly, the exogenous addition of netrin-1 to *Neo1*^−/−^ MEFs did not affect their pSmad1/5 response to low or high concentrations of BMP4 (Fig. 3a). Neogenin levels are increased in *Ntn1*-deficient mice^27^. Similarly, our *Ntn1*^−/−^ MEFs showed increased neogenin levels, as monitored by immunoblotting (Fig. 3b). *Lrig*-null MEFs showed a less pronounced increase in neogenin levels after *Ntn1* ablation than *Lrig* wild-type MEFs (Fig. 3b). To investigate whether the neogenin levels were regulated by BMP signaling per se, MEFs were treated with a BMP4 concentration that induces a robust pSmad1/5 response (10 ng/ml for 60 min) followed by immunoblotting. Clearly, this BMP4 stimulus did not affect the apparent neogenin levels, as determined through immunoblotting (Fig. S5e). The exogenous addition of recombinant netrin-1 to *Ntn1*^−/−^ MEFs resulted in a dose-dependent (Fig. 3c) and time-dependent (Fig. 3d) decrease in neogenin levels in both *Lrig* wild-type and *Lrig*-null MEFs. The induced expression of human LRIG1 or LRIG3 but not LRIG2 in *Ntn1* wild-type MEFs resulted in increased neogenin levels (Fig. 3e–g). This result was recapitulated in *Ntn1*^−/−^ MEFs with regard to the effect of LRIG1 but not LRIG3; i.e., the *Ntn1*^−/−^ MEFs showed significantly enhanced neogenin expression in response to the induced expression of LRIG1 (Fig. S6a) but not in response to the induced expression of LRIG3 (Fig. S6b).

### Netrin-1 suppresses adipogenesis and chondrogenesis in vitro

**Figure 4:**
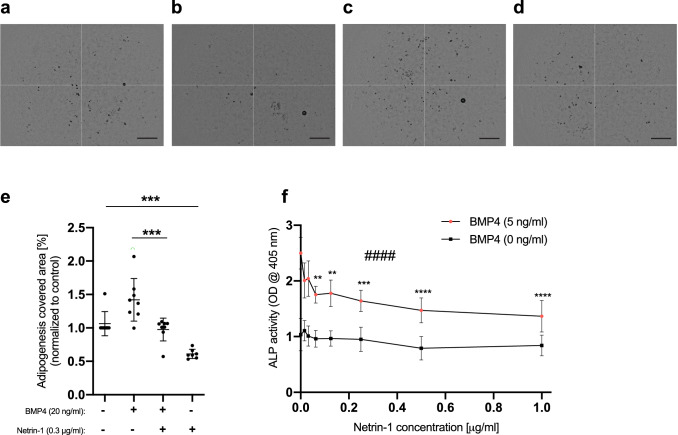
Netrin-1 inhibits adipogenesis and chondrogenesis in vitro. (**a**–**e**) Netrin-1 inhibits MEF adipogenesis. Wild-type MEFs were induced with an adipogenic cocktail together with or without 20 ng/ml BMP4 and/or 0.3 µg/ml netrin-1. After nine days, the cells were fixed, stained with Oil Red O, and imaged, and the staining was quantified. (**a**–**d**) Representative micrographs of MEFs treated with the adipogenic cocktail together with (**a**) no further additions, (**b**) BMP4, (**c**) BMP4 and netrin-1, and (**d**) netrin-1. Scale bars, 0.6 mm. (**e**) Quantification of the Oil Red O-stained areas as outlined in a-d. Shown are the means and individual values of eight independent experiments. (Student’s *t*-test: ***p < 0.001). (**f**) Netrin-1 inhibits chondrogenesis. ATDC5 cells were stimulated with BMP4 alone or in combination with different concentrations of netrin-1 for 72 h. Thereafter, chondrogenesis was assessed via an alkaline phosphatase (ALP) assay. The graph represents the average means with standard deviations of four independent experiments performed in duplicate. (One-way ANOVA: ^####^p < 0.0001. Post-hoc test with multiple comparisons [compared to netrin-1, 0 ng/ml] adjusted p-value: **p < 0.01; ***p < 0.001; ****p < 0.0001).

To investigate whether netrin-1 could regulate physiological processes in which BMP signaling plays important roles, we chose to study adipogenesis and chondrogenesis in vitro because these processes have been shown to depend on BMP signaling, albeit to our knowledge, the role of netrin-1 has not been addressed previously. Clearly, exogenously added recombinant netrin-1 was able to inhibit adipogenesis of MEFs as assessed through Oil Red O staining, both in the presence and in the absence of exogenously added BMP4 (Fig. 4a–e). Of note, also in the absence of added BMP4, the cell culture medium contained low levels of BMPs that are present in FBS, which is enough to drive basal adipogenesis^26,37,38^. Also chondrogenesis of BMP4-stimulated ATDC5 cells as assessed with an alkaline phosphatase activity assay was inhibited by netrin-1 (Figs. 4f and S8). As a control, the well-characterized BMP inhibitor noggin also inhibited BMP4-induced chondrogenesis of ATDC5 cells (Fig. S8b).

## Discussion

Despite the large number of investigations addressing the functions of netrins, to the best of our knowledge, a role of netrins in the regulation of BMP signaling has not been proposed previously. Our discovery showing that netrin-1 negatively regulated BMP signaling in a variety of cell types may explain many previous observations regarding the developmental and physiological functions of this protein.

Based on our discovery that netrin-1 inhibited BMP signaling, including via a mutually inhibitory interaction with the BMP-promoting activities of LRIG1 and LRIG3, together with previous work on inner ear development^5,6,28^, we propose that the molecular function of netrin-1, as well as the molecular functions of Lrig1 and Lrig3, in semicircular canal formation is to fine-tune BMP signaling. Similarly, the observed inhibition of adipogenesis and chondrogenesis in vitro is consistent with a function of netrin-1 in modulating BMP signaling during cell differentiation. Taken together, our results suggest that the regulation of BMP signaling might be a prominent feature of the developmental and physiological functions of netrin-1 and, possibly, other netrins.

Interestingly, we observed a commonality in the specificities of the BMP signal-modulating activities of netrin-1 and LRIG proteins. That is, netrin-1 suppressed the signaling that was induced by BMP4 and BMP6 but not the signaling that was induced by BMP9; these results reflect the BMP-promoting functions of Lrig proteins, which extend to BMP4 and BMP6 but not to BMP9^26^. This commonality in BMP ligand specificity may indicate that netrin-1 and LRIG proteins act through a common molecular machinery. However, our observation that netrin-1 and LRIG proteins also regulated BMP signaling in the absence of their mutually inhibitory counterparts suggests that netrin-1 and LRIG proteins function autonomously of each other. To resolve the underlying mechanisms, it will be important to elucidate the composition of the molecular machinery involved.

Regarding the netrin receptor(s) involved, we found that neogenin was required for the BMP inhibitory function of netrin-1 in MEFs. Neogenin is a well characterized receptor for netrin-1 as well as a coreceptor, together with the repulsive guidance molecules, for BMPs^29,30^. Hence, neogenin is well poised to integrate netrin and BMP signaling. Although the effect of netrin-1 on BMP signaling in MEFs was strictly dependent on neogenin, a role for other netrin receptors in the regulation of BMP signaling in MEFs or other cell types cannot be ruled out. For example, the neogenin homolog DCC is not expressed in MEFs (unpublished observation) but may play a role similar to neogenin in other cell types. UNC5B has been shown to activate BMP signaling during osteogenic differentiation^31^, although a possible regulatory role of netrins in this context was not investigated. Another study showed that two out of three netrin receptors (neogenin, Unc5b, and/or A2b) have to be inactivated to rescue MC3T3-E1 cells from netrin-1-induced inhibition of BMP4-induced osteoblast differentiation^7^, further supporting a connection between netrin-1 and BMP signaling as well as demonstrating a functional redundancy among the netrin receptors. Future studies will reveal the roles of different netrin receptors in the regulation of BMP signaling. In parallel to its inhibition of BMP signaling, netrin-1 downregulated neogenin protein expression levels both in a genetic manner, as evident from the upregulation of neogenin seen in *Ntn1*^−/−^ cells, as well as biochemically, as seen in the cellular response to recombinant netrin-1 protein. These observations are consistent with previous reports showing that *Ntn1*^−/−^ mice display increased neogenin and DCC levels^27^ and that exogenous netrin-1 downregulates the DCC levels in embryonic cortical neurons^32^. In contrast, LRIG proteins upregulated the neogenin levels in parallel with their enhancement of BMP signaling. This is consistent with a previous report that showed a physical interaction between Lrig proteins and neogenin, which was associated with the protection of neogenin from proteolytic cleavage by ADAM17^33^. To what degree the netrin-1- and LRIG-mediated effects on neogenin expression levels represent causes or consequences of signal regulation remains to be investigated. In this regard, it is intriguing that all three Lrig proteins (Lrig1-3) seem to protect neogenin from proteolytical degradation^33^, whereas only LRIG1 and LRIG3, but not LRIG2, promote BMP signaling^26^.

Several of the netrin receptors, including DCC, neogenin, UNC5A, UNC5B, UNC5C, and UNC5D, have been proposed to function as so-called ‘dependence receptors’, i.e., receptors that mediate pro-apoptotic signals in the absence of ligand. Here, we propose an alternative hypothesis to account for some of the published observations pertaining to this concept. We suggest that in the absence of their netrin ligands, dependence receptors may promote basal BMP signaling, as has been shown for neogenin and UNC-40^30,34,35^, rather than exerting ‘ligand-independent’ signaling. Constitutive low-level BMP signaling is possible because different BMPs are widely expressed in mammalian tissues and are also present in FBS-containing cell culture media^36,37^. Then if, as shown here, netrin-1 inhibits BMP signaling at low BMP concentrations, we propose that some of the cellular effects caused by netrin-1 may be due to its negative regulation of BMP signaling rather than, or in addition to, its interruption of the intrinsic ligand-independent signaling of dependence receptors.

Netrin-1 has also been reported to be overexpressed and to promote cancer in many other contexts. We noticed an intriguing reciprocal relationship between the reported effects of netrin-1 and BMP signaling on several types of cancer cells. For example, although netrin-1 promotes the development, spread, metastasis, and stemness of breast cancer, colorectal cancer, and glioma cells, BMP signaling reciprocally suppresses these cancer cell types and processes, at least under some circumstances^38–42^. Thus, our results presented herein, which reveal a previously unrecognized function of netrin-1 as a suppressor of BMP signaling, are consistent with a tumor-promoting function of netrin-1 that is achieved via the suppression of BMP signaling. This function may warrant further investigation.

In conclusion, our demonstration that netrin-1 regulates BMP signaling via its receptor neogenin sheds new light on previous observations and opens up new areas for research. The urgent questions to address include the elucidation of the detailed molecular mechanisms involved and the delineation of the physiological processes that are directed by this regulatory mechanism.

## Supplementary Information

Supplementary Information.

## Abbreviations

BMP: Bone morphogenetic protein
FBS: Fetal bovine serum
LRIG: Leucine-rich repeats and immunoglobulin-like domains
MEF: Mouse embryonic fibroblast
PBS: Phosphate-buffered saline
