# Supplementary material for: Netrin-1 functions as a suppressor of bone morphogenetic protein (BMP) signaling
Source: Sci Rep. 2021 Apr 21;11:8585. doi: 10.1038/s41598-021-87949-7 (PMC8060280; doi:10.1038/s41598-021-87949-7)

SUPPLEMENTARY INFORMATION

Netrin-1 functions as a suppressor of bone morphogenetic protein (BMP) signaling

Ahmad Abdullah[†,1], Carl Herdenberg[†,1], and Håkan Hedman[1,*]

[†]These authors contributed equally to the work
[1]Department of Radiation Sciences, Umeå University, SE-90187 Umeå, Sweden

***Correspondence**
H. Hedman, Oncology Research Laboratory, NUS M31, SE-90185 Umeå, Sweden
Tel: +46-90-785 2881
E-mail: hakan.hedman@umu.se

SUPPLEMENTARY INFORMATION

Figure legends

Supplementary Figure S1. Western blot analyses of *Ntn1*-deficient and control MEF lines. Netrin-1 expression was knocked out in wild-type MEFs or *Lrig*-null MEFs with inducible *LRIG1* or *LRIG3* alleles using CRISPR/Cas9 followed by cell cloning. Protein expression was evaluated via Western blotting using antibodies against netrin-1, the FLAG epitope present on the inducible LRIG1 and LRIG3 proteins, or actin as the loading control. (**a**) Blots showing wild-type MEFs (ScA1, ScA3, ScC3, and ScD2) and *Ntn*$^{-/-}$;*Lrig*-wild-type MEFs (G2B2, G2A2, and G2C1). (**b**) Blots showing LRIG1-inducible *Ntn*$^{-/-}$ MEFs that had not been induced (-dox) or had been induced (+dox) to express LRIG1. (**c**) Blots showing LRIG3-inducible *Ntn*$^{-/-}$ MEFs that had not been induced (-dox) or had been induced (+dox) to express LRIG3. Uncropped blots are shown in Figure S9.

Supplementary Figure S2. Receptor levels in MEFs of different *Lrig* and *Ntn1* genotypes. (**a-c**) Expression levels of Bmpr2, Acvr1, and Smad1 in MEFs of different *Ntn1* (*Ntn1*$^{+/+}$ or *Ntn1*$^{-/-}$) and *Lrig* (*Lrig*-wild-type or *Lrig*-null (*Lrig1*$^{-/-}$*; Lrig2*$^{-/-}$*; Lrig3*$^{-/-}$) genotypes analyzed by Western blotting. Graphs represent average means with standard deviations of band intensity ratios (neogenin/actin) from four *Ntn1*$^{+/+}$*;Lrig*-wild-type or *Ntn1*$^{+/+}$*;Lrig*-null biological replicates and three *Ntn1*$^{-/-}$*;Lrig*-wild-type or *Ntn1*$^{-/-}$*;Lrig*-null biological replicates that were determined with three independent experiments. (**d**) Representative blots of the four clones of wild-type and three clones each of *Lrig*-wild-type/*Ntn1*$^{-/-}$ and *Lrig*-null/*Ntn1*$^{-/-}$, respectively.

Supplementary Figure S3. Lrig and netrin-1 expression levels in MEFs of different *Ntn1* and *Lrig* genotypes. (**a-d**) Expression of Lrig proteins in wild-type and *Ntn1*-deficient MEF lines. (**a, c**) Representative blots showing Lrig1 and Lrig3 expression in four clones of wild-type MEFs and three clones of *Ntn1*-deficient MEFs. (**e**) Representative blot showing netrin-1 expression in four clones each of wild-type and *Lrig*-null MEF lines. (**b, d, f**) Graphs showing the average means with standard deviations of four independent experiments. Uncropped blots are shown in Figure S10.

Supplementary Figure S4. Full-length blots. Shown are the full-length blots corresponding to the cropped versions shown in Figure 2.

Supplementary Figure S5. Western blot analyses of wild-type and *Neo1*-deficient MEF lines. (**a**) Representative blots showing the expression of neogenin, netrin-1, Lrig1, Lrig3, and actin as loading control in four clones of *Neo1* wild-type (S1, S2, S3, and S4) and *Neo1*-deficient (1A1, 1B1, 1B2, and 2C4) MEF lines. Uncropped blots are shown in Figure S11. (**b-d**) Graphs representing average means with standard deviations for the expression of Lrig1, Lrig3, and netrin-1 in *Neo1* wild-type and *Neo1*-deficient MEF lines from four biological repeats. (e) Neogenin expression in wild-type MEFs treated or not treated with 10 ng/ml

BMP4 for 60 minutes. Shown are the average means and standard deviations from four independent experiments.

Supplementary Figure S6. Neogenin levels in LRIG1- and LRIG3-inducible *Ntn1*-deficient MEFs. Effects of induced expression of LRIG1 (**a**) and LRIG3 (**b**) on neogenin expression levels in *Ntn1*-deficient MEFs. LRIG expression was induced through the treatment of LRIG-inducible MEFs with 100 ng/ml doxycycline overnight. Neogenin and actin levels were analyzed using Western blotting. Representative blots and graphs representing average means with standard deviations of four independent experiments are shown. (Student's t-test; ***, $p<0.001$). Uncropped blots are shown in Figure S12.

Supplementary Figure S7. Full-length blots. Shown are the full-length blots corresponding to the cropped versions shown in Figure 3. Red squares indicate the parts that were displayed in Figure 3.

Supplementary Figure S8. Netrin-1 and noggin inhibit BMP4-induced ATDC5 cell chondrogenesis. ATDC5 cells were treated with (**a**) different concentrations of BMP4 in the absence or presence of netrin-1 (0.25 µg/ml) or noggin (10 ng/ml), or (**b**) different concentrations of noggin in the absence or presence of BMP4 (5 ng/ml) for 72 hours. Thereafter, chondrogenesis was assessed via an alkaline phosphatase (ALP) assay. Graphs represent the average means with standard deviations from four independent experiments. One-way ANOVA: ####, $p<0.0001$.

Supplementary Figure S9. Full-length blots. Shown are the full-length blots corresponding to the cropped versions shown in Figure S1. Red squares indicate the parts that were displayed in Figure S1.

Supplementary Figure S10. Full-length blots. Shown are the full-length blots corresponding to the cropped versions shown in Figure S3. Red squares indicate the parts that were displayed in Figure S3.

Supplementary Figure S11. Full-length blots. Shown are the full-length blots corresponding to the cropped versions shown in Figure S5. Red squares indicate the parts that were displayed in Figure S5.

Supplementary Figure S12. Full-length blots. Shown are the full-length blots corresponding to the cropped versions shown in Figure S6. Red squares indicate the parts that were displayed in Figure S6.

SUPPLEMENTARY INFORMATION

Tables

Table S1. Antibodies used in the study.

Antigen and antibody conjugate	Host	Company	Catalog no.	Lot no.	Application[a]	Dilution
Netrin-1	Rabbit	Abcam	ab126729	GR250605-25	WB	1:1,000
Neogenin	Rabbit	Novus Biologicals	NBP1-89651	A91773	WB	1:1,000
Bmpr2	Mouse	Fisher Scientific	3F6 F8	td269789	WB	1:500
ACVR1	Rabbit	Novus Biologicals	NBP1-33500	40142	WB	1:500
Smad1	Rabbit	Cell Signaling Technology	6944S	5	WB	1:1,000
Actin	Mouse	Cell Signaling Technology	3700	17	WB	1:5,000
pSmad1/5	Rabbit	Cell Signaling Technology	9516	9	WB, ICC	1:1,000 (WB), 1:800 (ICC)
FLAG M2	Mouse	Sigma-Aldrich	F3165	SLBN8915V	WB, ICC	1:20,000 (WB),1:2,000 (ICC)
Mouse IgG IRDye 800CW	Goat	LI-COR Biosciences	926-32210	C81106-03	WB	1:15,000
Rabbit IgG IRDye 680RD	Goat	LI-COR Biosciences	925-68071	C60606-01	WB	1:15,000
Alexa fluor anti-rabbit 647	Goat	Invitrogen	A21245	2098544	ICC	1:1,000
Alexa fluor anti-mouse 488	Donkey	Invitrogen	A21202	13053	ICC	1:1,000

[a]WB, Western blotting; ICC, immunocytochemistry

Supplementary Figure S1

a

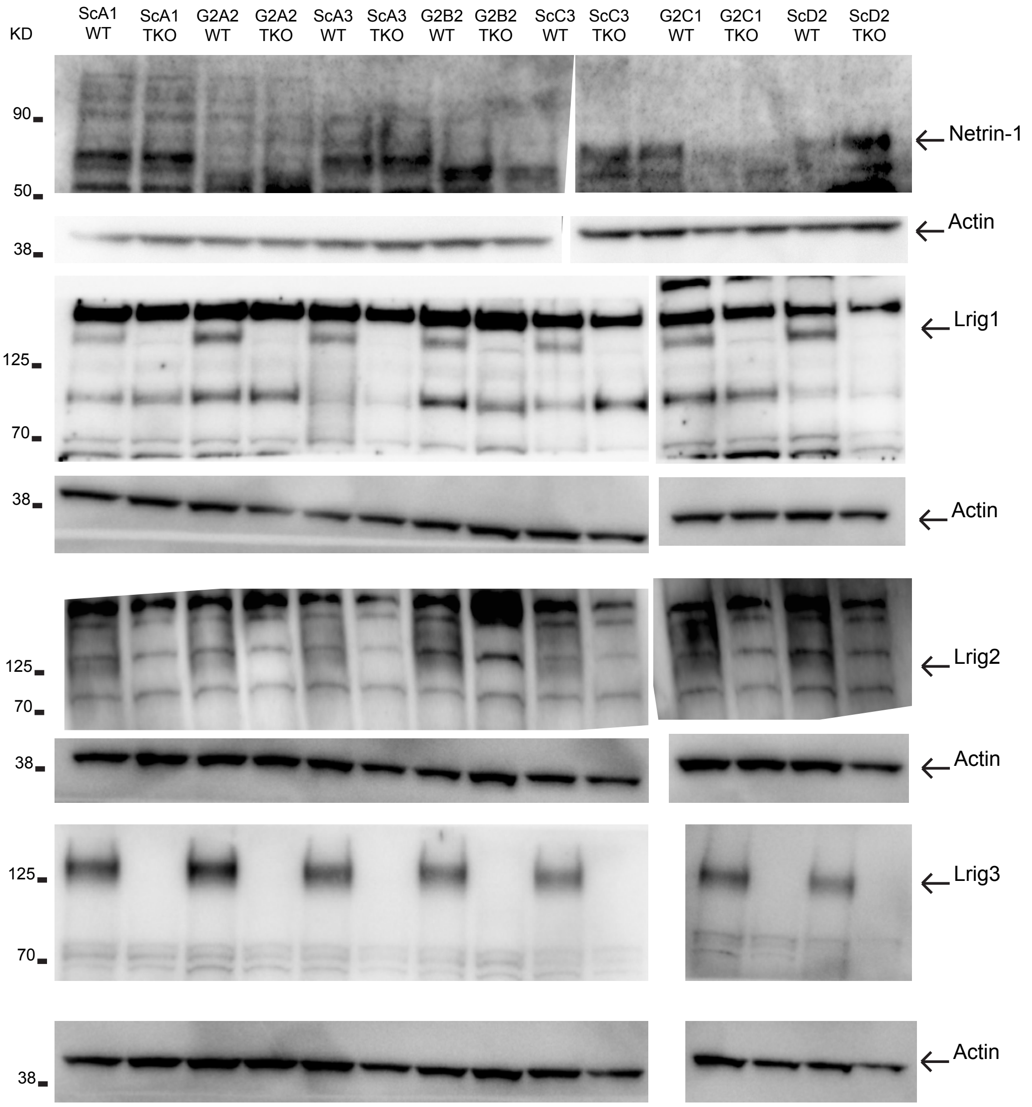

b

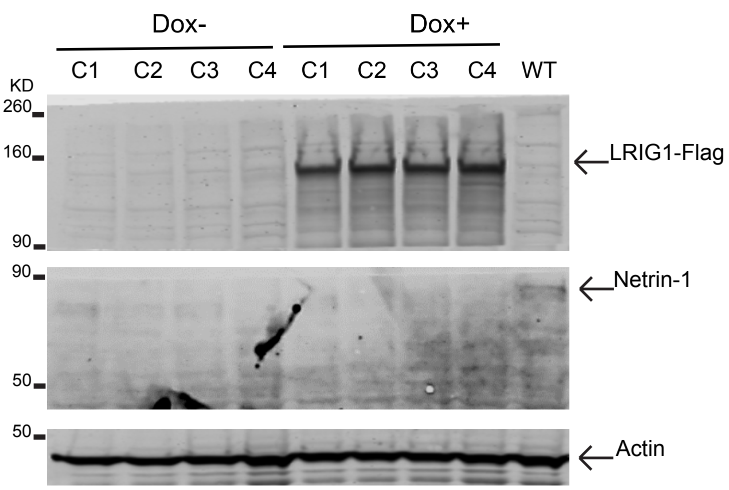

c

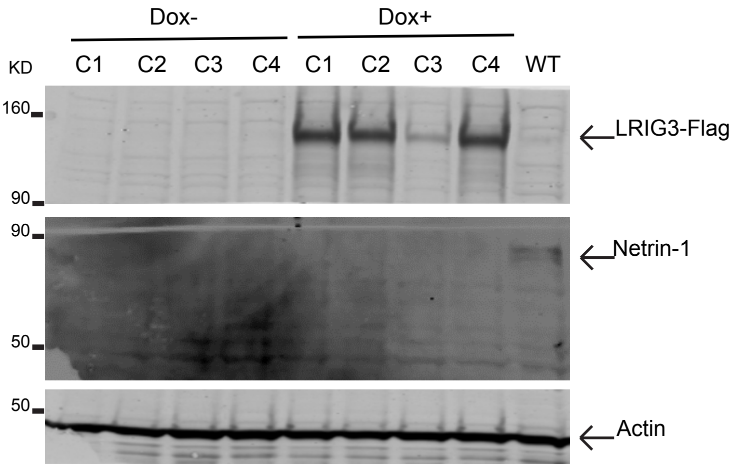

Supplementary Figure S2

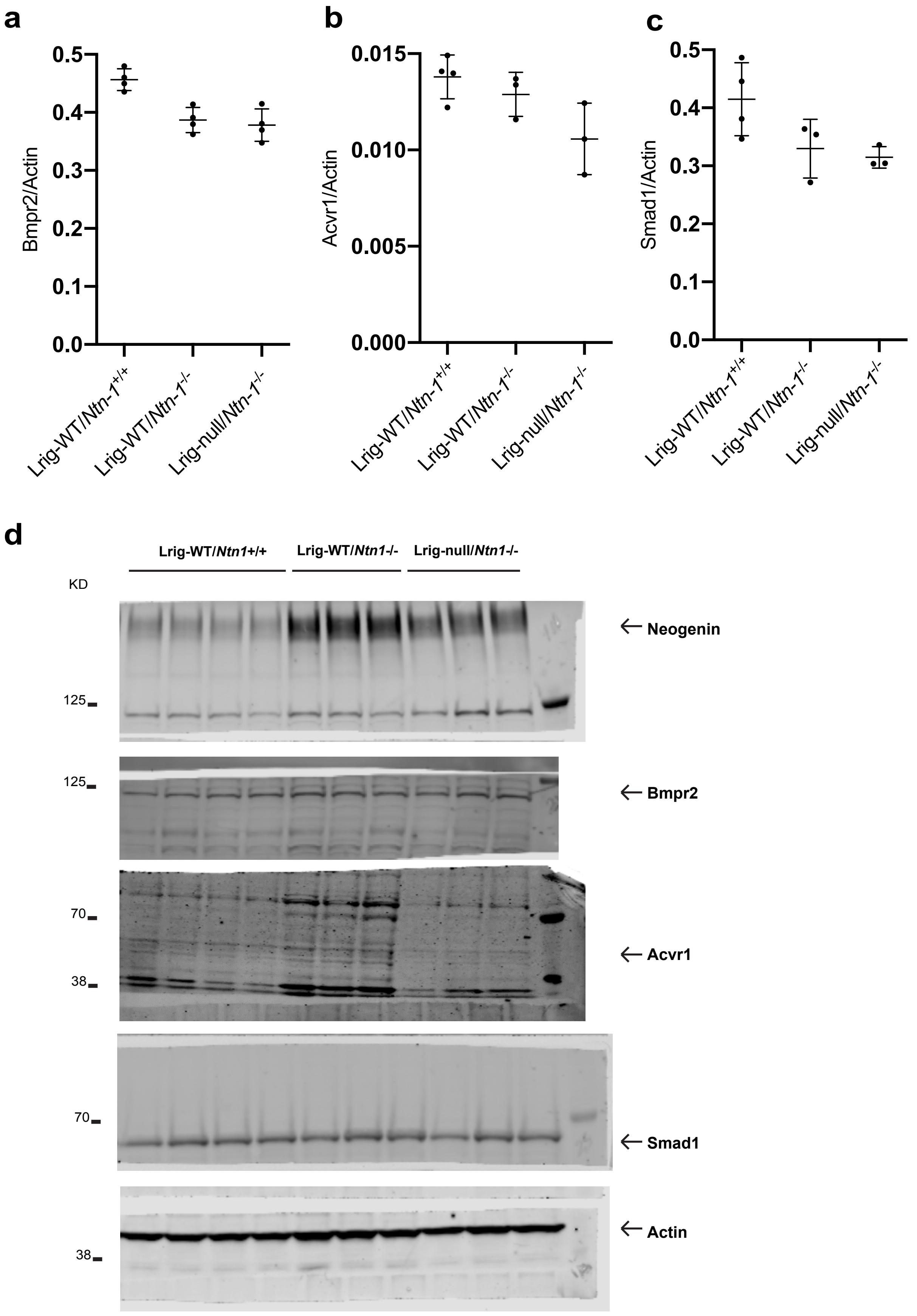

Supplementary Figure S3

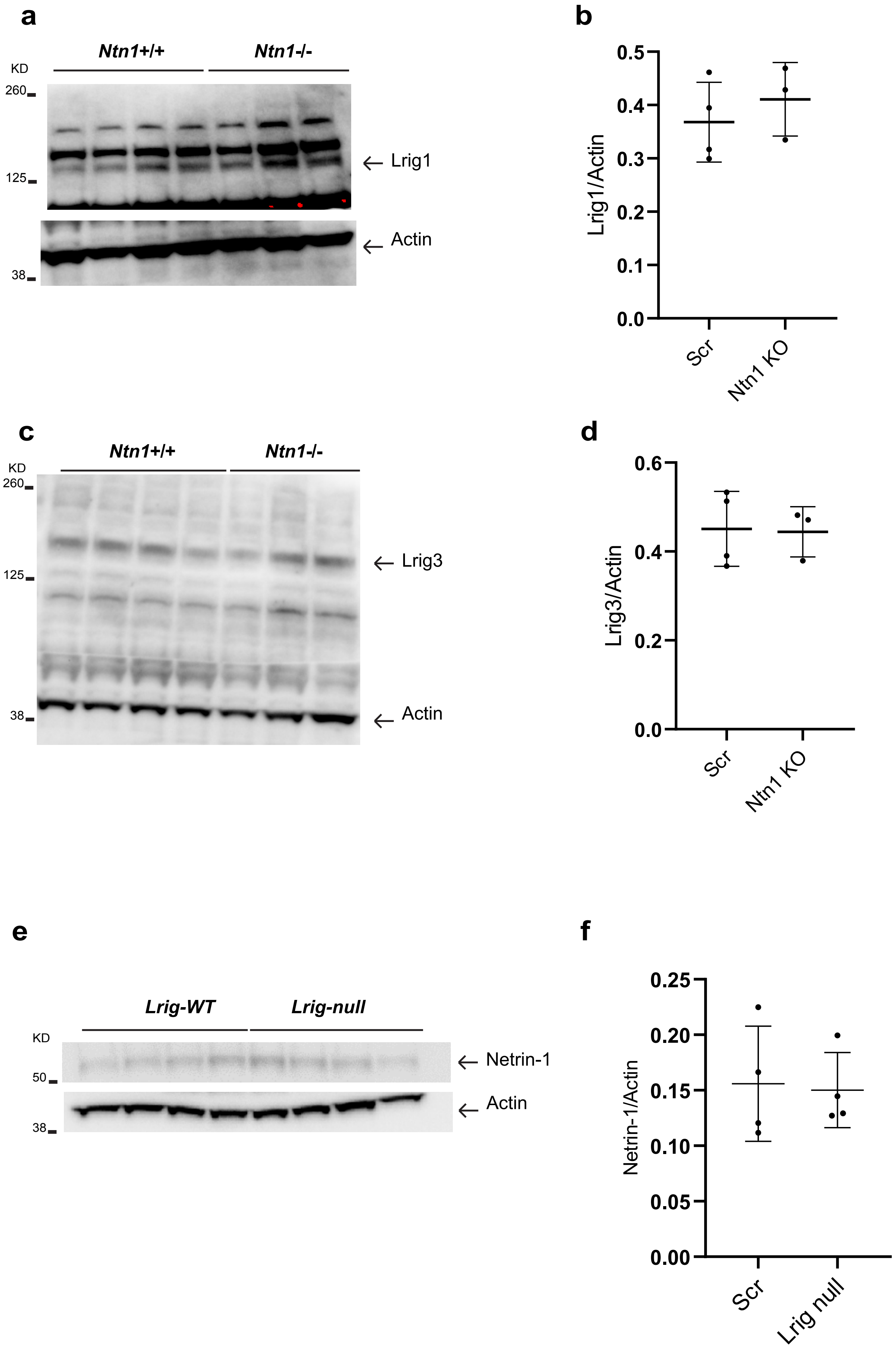

Supplementary Figure S4

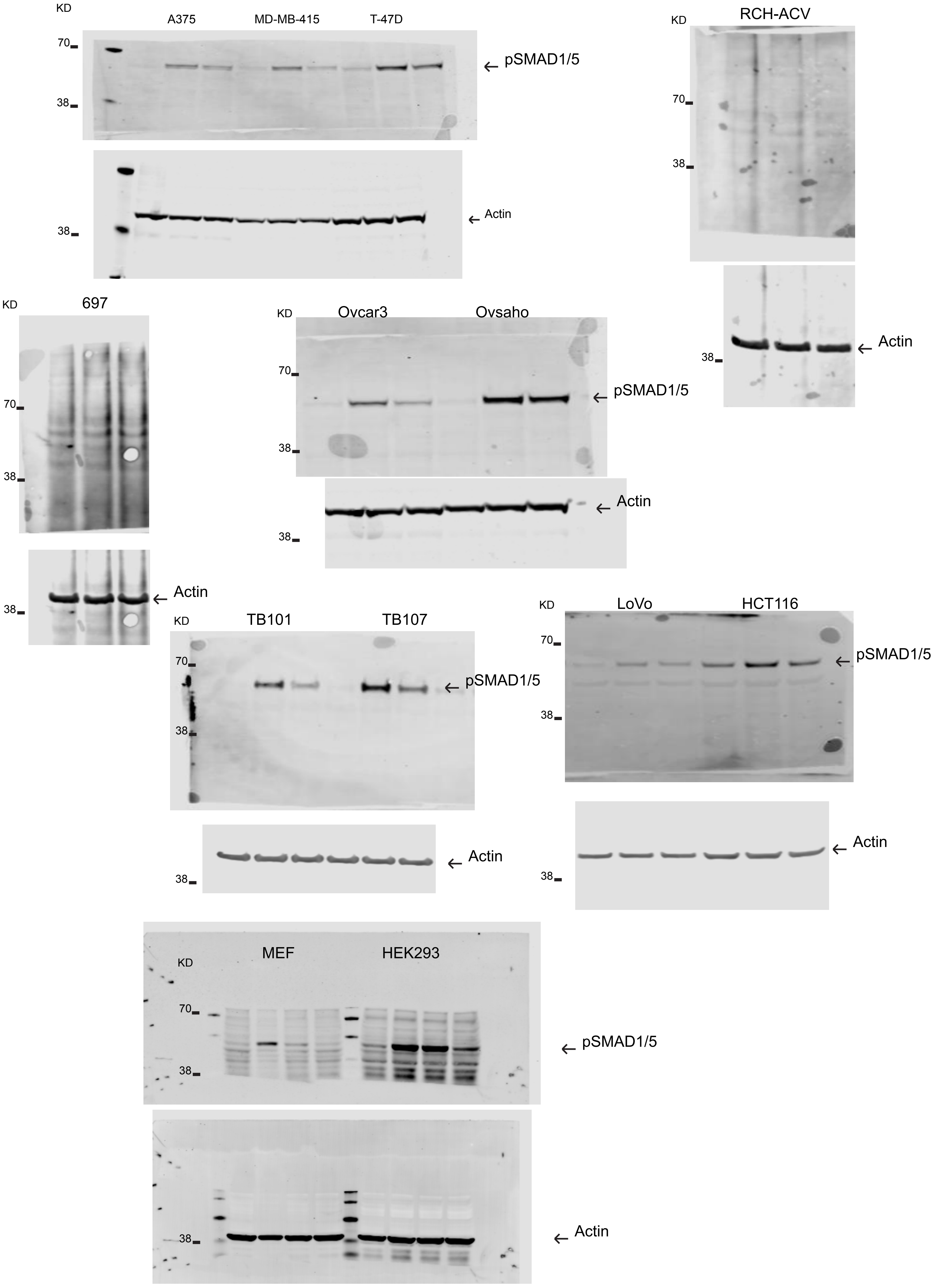

Supplementary Figure S5

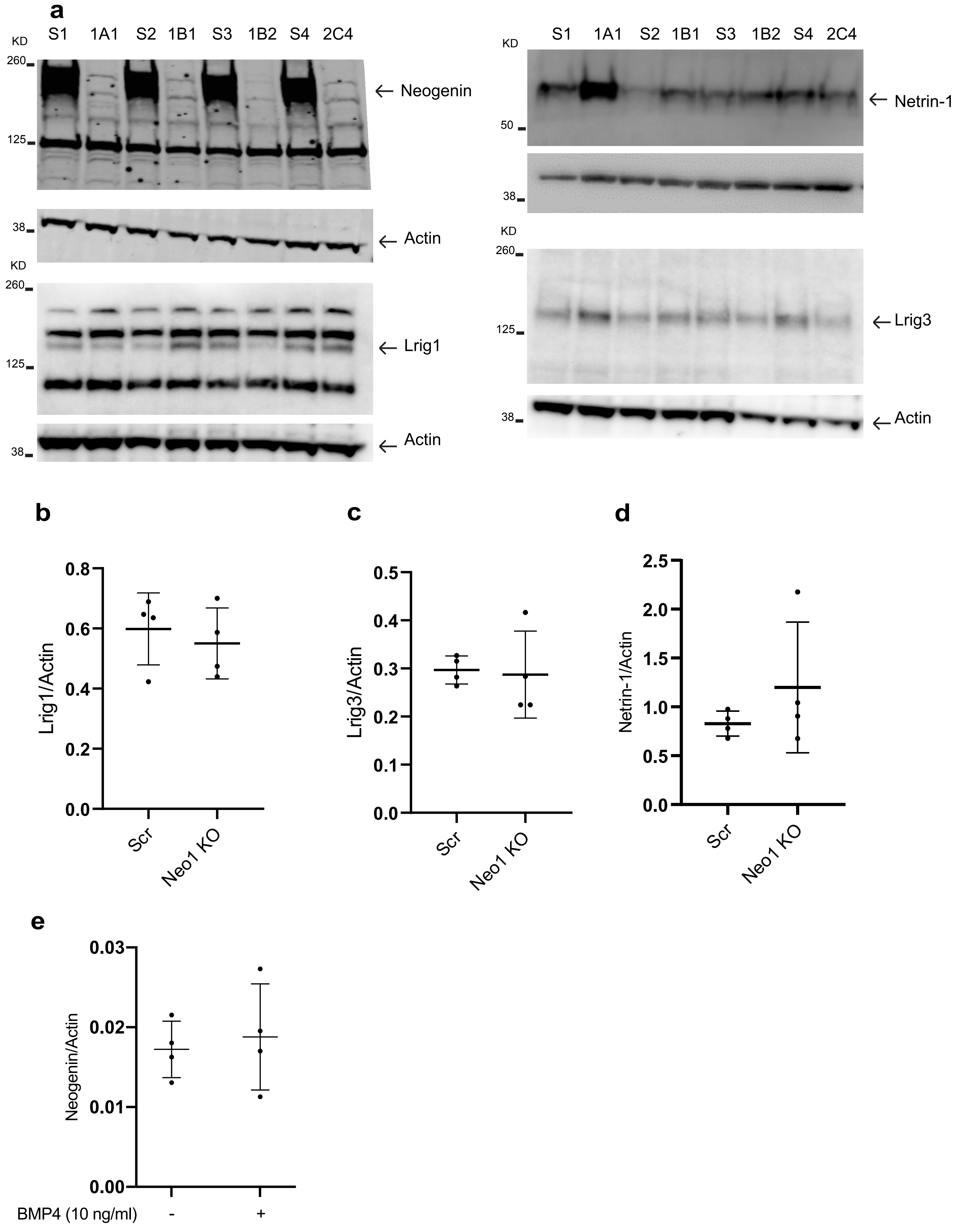

Supplementary Figure S6

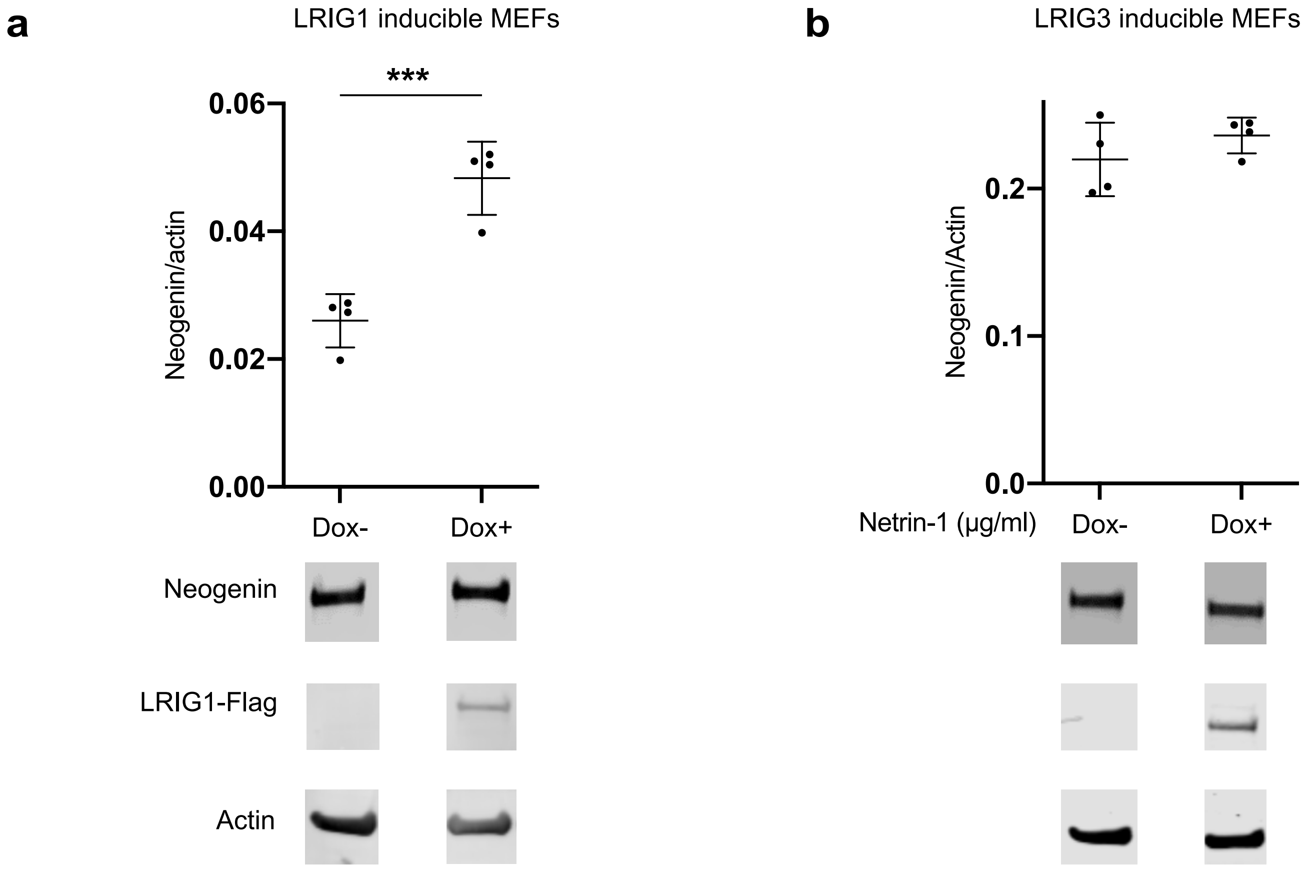

Supplementary Figure S7

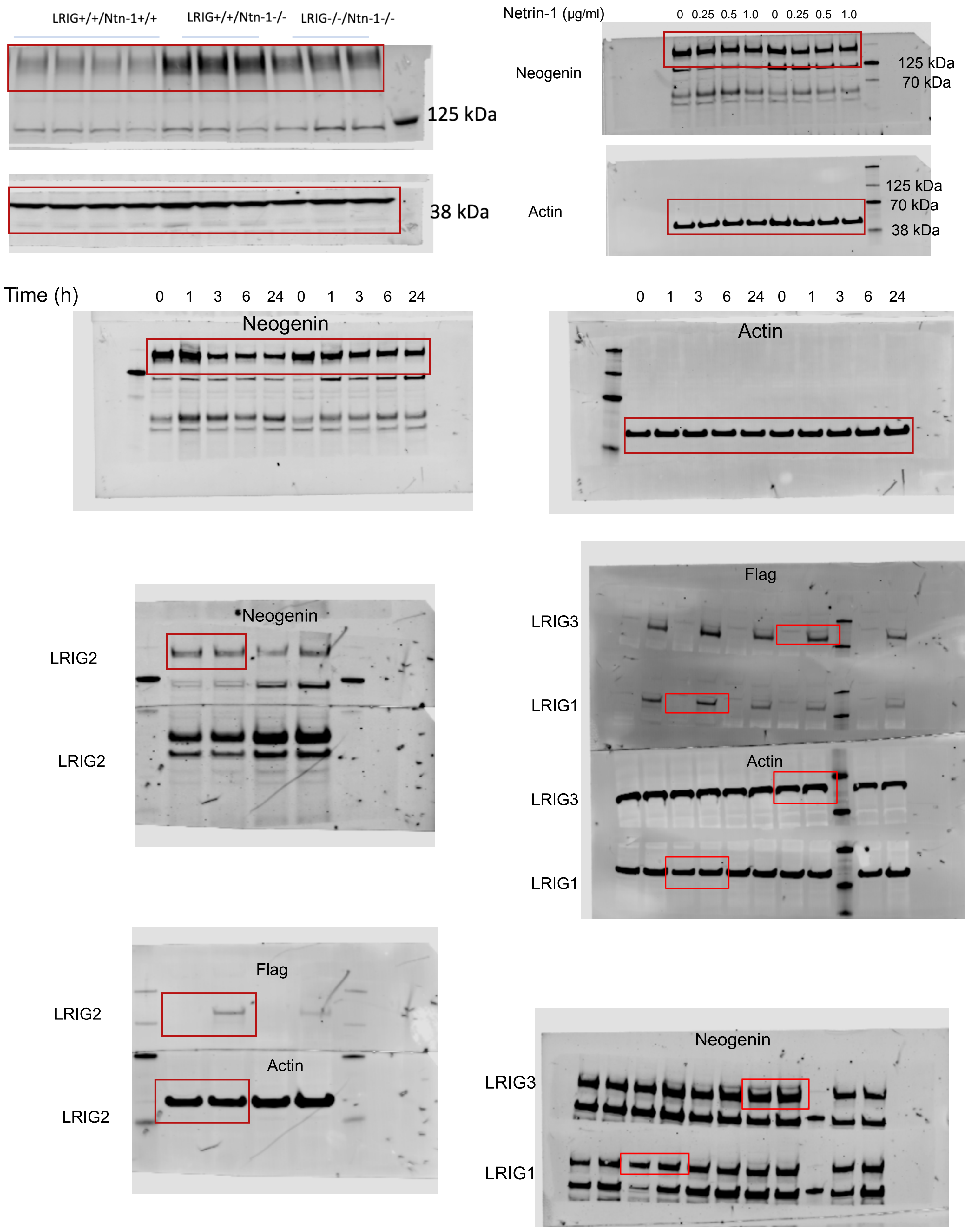

Supplementary Figure S8

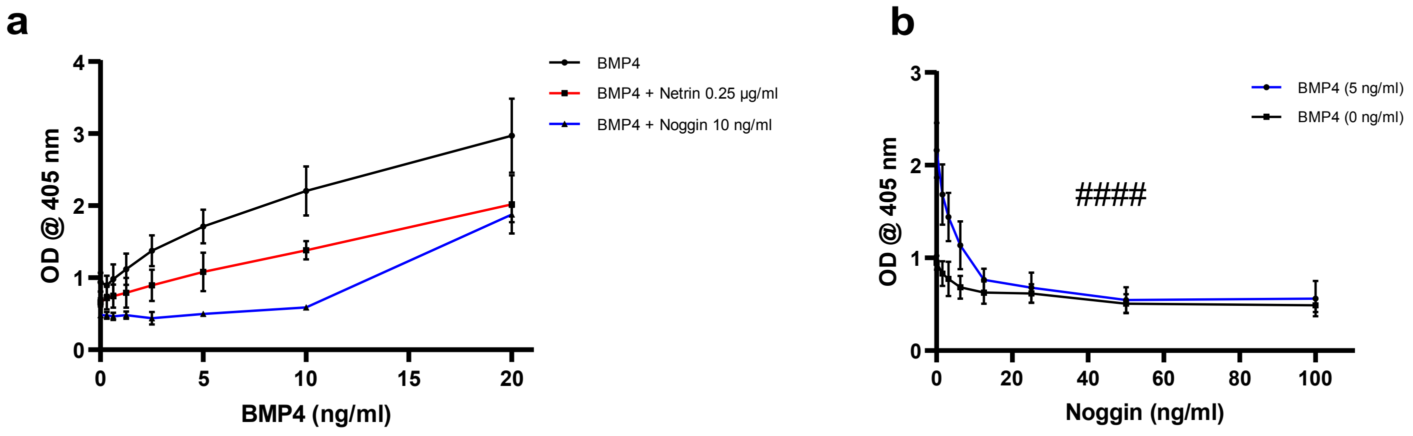

Supplementary Figure S9

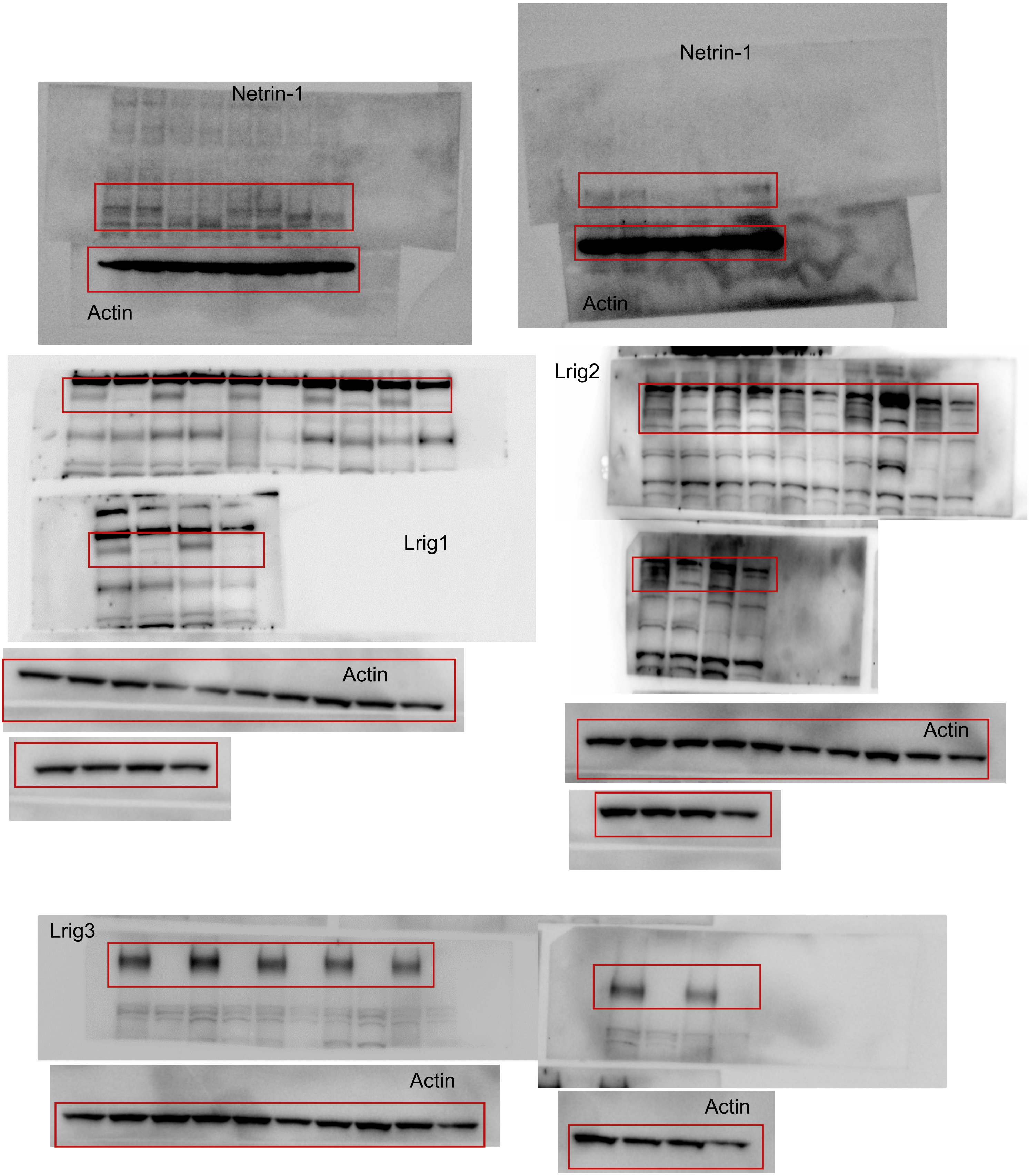

Supplementary Figure S10

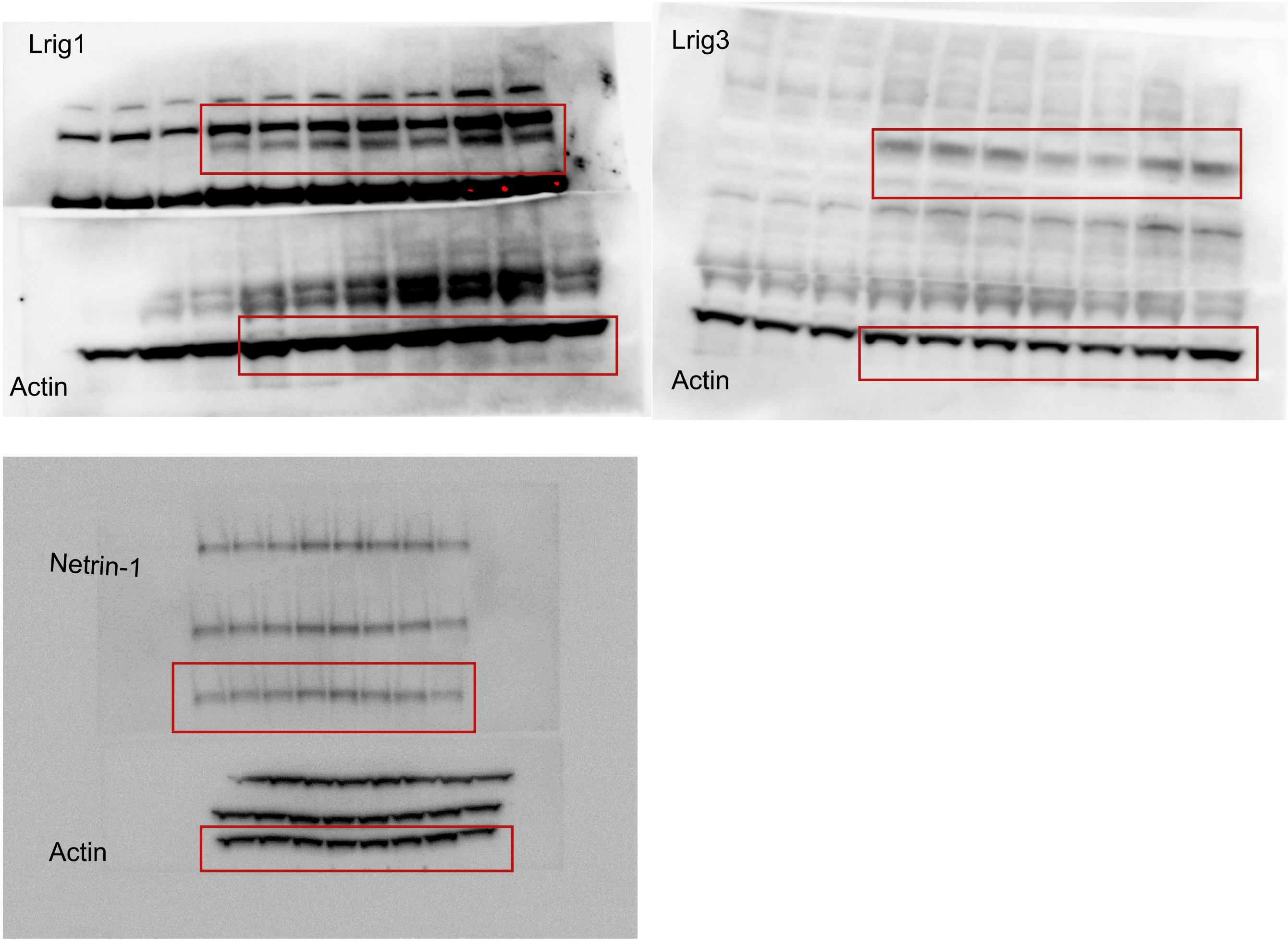

Supplementary Figure S11

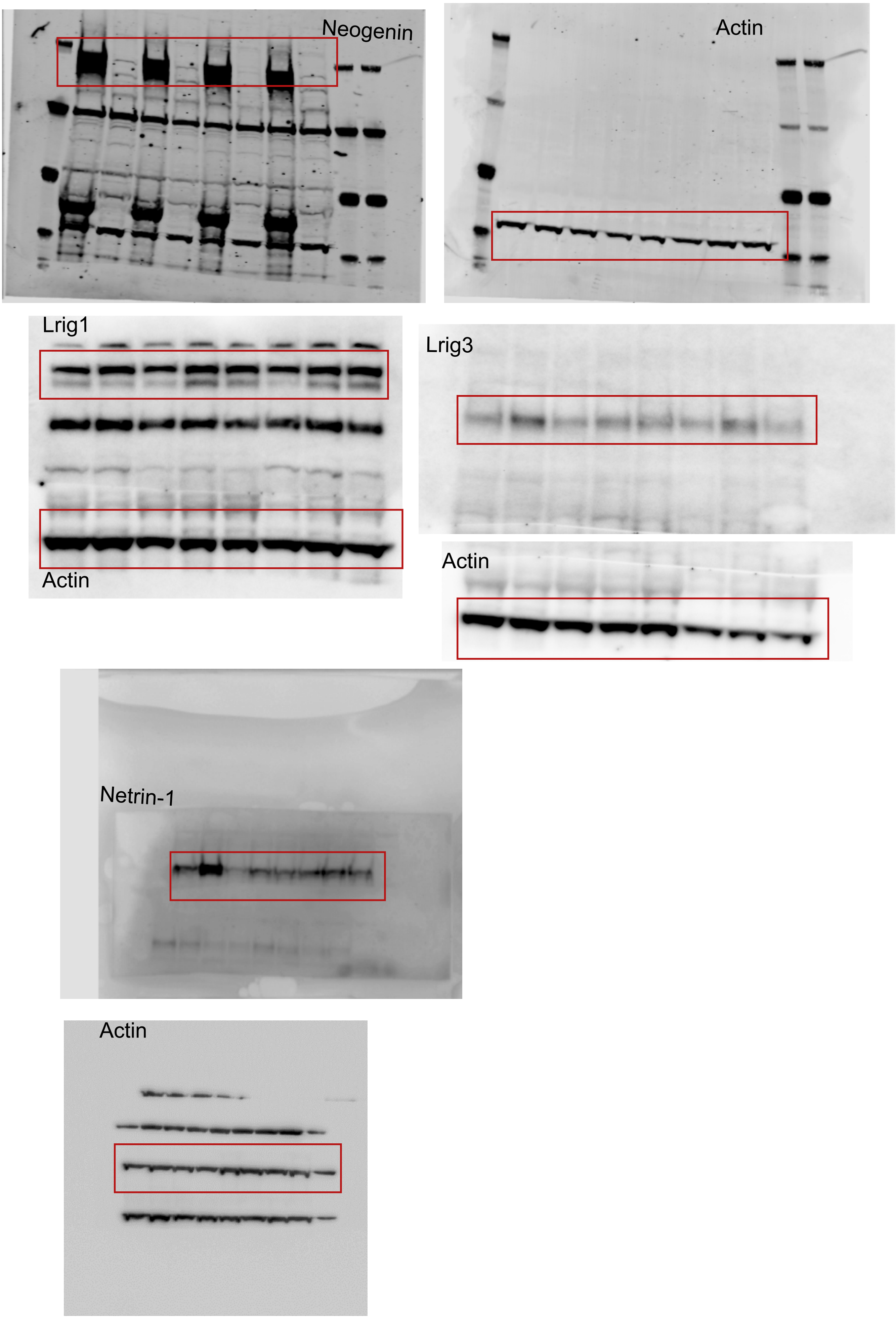

Supplementary Figure S12

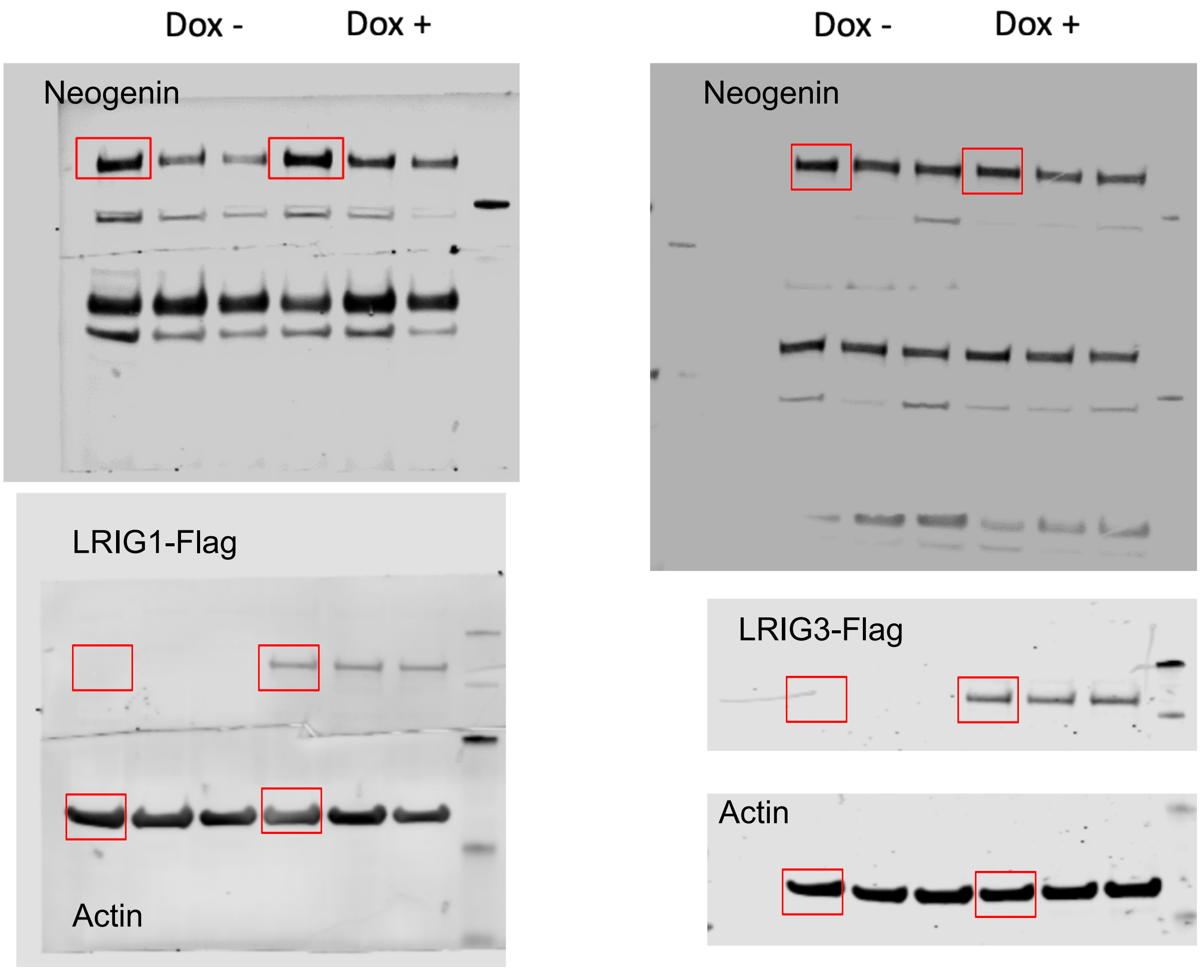

Supplement: Supplementary file 1 — Supplementary Information. [file 41598_2021_87949_MOESM1_ESM.pdf]
